# Antibody secreting cells are critically dependent on integrin α4β7/MAdCAM-1 for intestinal recruitment and control of the microbiota during chronic colitis

**DOI:** 10.1038/s41385-021-00445-z

**Published:** 2021-08-25

**Authors:** Christopher J. Tyler, Mauricio Guzman, Luke R. Lundborg, Shaila Yeasmin, Nadia Zgajnar, Paul Jedlicka, Giorgos Bamias, Jesús Rivera-Nieves

**Affiliations:** 1 San Diego VA Medical Center, San Diego, CA USA; 2 Division of Gastroenterology, University of California San Diego, La Jolla, CA USA; 3 Department of Pathology, University of Colorado Denver, Denver, CO USA; 4 GI Unit, 3rd Academic Department of Internal Medicine, Sotiria Hospital, National and Kapodistrian University of Athens, Athens, Greece

## Abstract

T and B cells employ integrin α4β7 to migrate to intestine under homeostatic conditions. Whether those cells differentially rely on α4β7 for homing during inflammatory conditions has not been fully examined. This may have implications for our understanding of the mode of action of anti-integrin therapies in inflammatory bowel disease (IBD). Here, we examined the role of α4β7 integrin during chronic colitis using IL-10^−/−^ mice, β7-deficient IL-10^−/−^, IgA-deficient IL-10^−/−^ mice, and antibody blockade of MAdCAM-1. We found that α4β7 was predominantly expressed by B cells. β7 deficiency and MAdCAM-1 blockade specifically depleted antibody secreting cells (ASC) (not T cells) from the colonic LP, leading to a fecal pan-immunoglobulin deficit, severe colitis, and alterations of microbiota composition. Colitis was not due to defective regulation, as dendritic cells (DC), regulatory T cells, retinaldehyde dehydrogenase (RALDH) expression, activity, and regulatory T/B-cell cytokines were all comparable between the strains/treatment. Finally, an IgA deficit closely recapitulated the clinical phenotype and altered microbiota composition of β7-deficient IL-10^−/−^ mice. Thus, a luminal IgA deficit contributes to accelerated colitis in the β7-deficient state. Given the critical/nonredundant dependence of IgA ASC on α4β7:MAdCAM-1 for intestinal homing, B cells may represent unappreciated targets of anti-integrin therapies.

## Introduction

Maintenance of a homeostatic microbiota is critically dependent on antibody secreting cells (ASC) which release IgA to maintain a balance between the microbiota and its host^1^. Loss of tolerance to antigens derived from the intestinal microbiota has been implicated in the pathogenesis of inflammatory bowel disease (IBD). Yet little effort has been placed on understanding the role of B cells/IgA during the pathogenesis of IBD, particularly considering that about 80% of the body’s ASC are found in the intestine. Lymphocytes in general and B cells in particular are imprinted with a gut-homing program within inductive sites (e.g., Peyer patches (PP), mesenteric lymph nodes (MLN)) by upregulating α4β7 integrin expression mediated by retinoic acid (RA)-producing dendritic cells (DC)^2^. This enables homing to the intestine and gastrointestinal-associated lymphoid tissues (GALT) through specialized venules that express the α4β7 ligand: mucosal addressin cell adhesion molecule-1 (MAdCAM-1). MAdCAM-1 is an endothelial adhesion molecule of the immunoglobulin superfamily expressed by postcapillary venules of the small and large intestinal lamina propria (LP), MLN, and mammary gland^3^.

A critical dependence of B cells/ASC on α4β7/MAdCAM-1 interactions for intestinal homing is supported by the fact that IgA^+^ ASC are decreased in both β7- and MAdCAM-1-deficient mice^4,5^. However, whether there may be differential dependence by T or B cells for intestinal recruitment has not been addressed. This is particularly relevant to understand the mode of action of drugs that targets this family of integrins in IBD, which is hallmarked by chronic dysregulated leukocyte recruitment. The integrin-antagonist vedolizumab^6,7^ (VDZ, anti-α4β7) is widely used for the treatment of IBD. Two additional drugs, etrolizumab (anti-β7) and ontamalimab (anti-MAdCAM-1), are undergoing clinical trials^8^. B cells and ASC express α4β7^9^ and bind to the anti-α4β7 antibody VDZ at equal or higher levels than T cells^10^. A commonly held premise is that VDZ is intestinal specific, and acts mainly by blocking effector T-cell entry to the intestinal LP. However, there is insufficient evidence for this from human data^11^, where minimal effect on LP T-cell populations has been observed, implicating innate immune cells, namely macrophages and DC^12^ and monocytes^13^. Clinically, less than 50% of IBD patients who are treated with VDZ achieve long-term clinical remission, and an even smaller fraction meets the more demanding treatment targets of mucosal, histological or deep remission^6,7^. These shortcomings emphasize the need to clearly define its mechanism of action to better identify patients with increased probability for response^14^. In fact, despite widespread use of VDZ for more than 6 years, the precise cell targets which define its anti-inflammatory effects remain uncertain^11,14^.

We set out to evaluate the immune cell lineages most reliant on integrin α4β7 to enter the intestine during chronic inflammatory conditions, using the IL-10^−/−^ murine model of chronic colitis. IL-10 deficient mice develop spontaneous colitis that shares features with ulcerative colitis (UC), marked by epithelial cell hyperplasia and transmural inflammation. The microbiota plays a major role in this model^15^, as IL-10^−/−^ germ-free mice do not develop colitis, whereas colonization by *Helicobacter hepaticus* triggers colitis^16^*.* We observed dichotomous α4 integrin expression by T and B cells, inasmuch the former express predominantly α4β1, and the latter α4β7. CD3^+^ cells increased in colonic LP of β7-sufficient and deficient colitic mice compared with noninflamed controls, whereas B cell/ASC populations did not change in β7-deficient mice. The B cell/ASC deficit in the LP resulted in a luminal pan-immunoglobulin deficit and severe colitis, compared with β7-sufficient mice. Colitis was not due to defective immune regulation, as DC, regulatory T cells (Tregs), RALDH expression, RALDH activity, and regulatory T/B-cell cytokines were all comparable between the strains. Furthermore, MAdCAM-1 blockade, which does not interfere with αEβ7:E-cadherin interactions, impaired B cell/ASC recruitment specifically and recapitulated the colitic phenotype of β7-deficient animals in real time. Finally, we identified IgA as the molecular effector for the accelerated colitis in the β7-deficient state as IgA deficiency closely recapitulated the severe colitic phenotype, bacterial overgrowth, and alterations in fecal microbiota composition.

## Results

### B cells preferentially express integrin α4β7

**Fig. 1:**
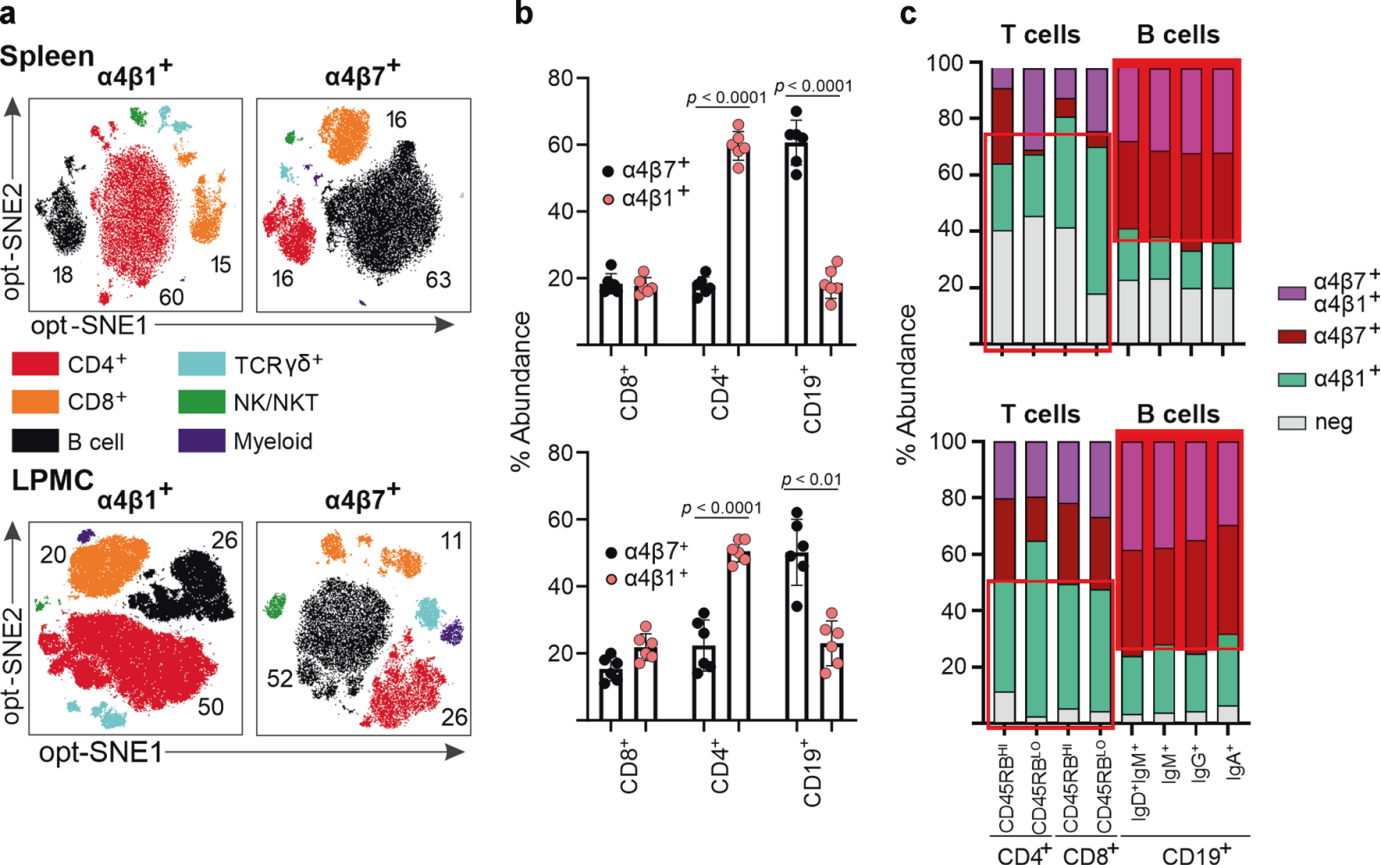
B cells predominantly express α4β7 integrin. **a** Cellular distribution of integrins α4β1 and α4β7 among the major leukocyte lineages within the peripheral (spleen) and mucosal (colonic LP) compartments. Cells were pregated on live, CD45^+^ cells, followed by opt-SNE analysis. Cell populations including CD4^+^ (CD4^+^ T cells), CD8^+^ (CD8^+^ T cells), CD19^+^/IgA^+^/IgG^+^ (B cells), TCRγδ^+^ (γδ T cells), CD56^+^ (NK/NKT), or MHCII^+^/CD11c^+^/CD11b^+^ (myeloid) cells are highlighted. **b** Percentages of α4β1- and α4β7-expressing cells within the indicated cell lineages from colonic LPMC and splenocytes. Each data point represents a single mouse. All data are presented as mean ± S.D, from *n* > 6 mice in each dataset. Statistical significance determined using ANOVA, followed by Tukey’s multiple comparison test. **c** Distribution of integrin expressing subpopulations within the CD4^+^, CD8^+^, and B-cell lineages. T- and B-cell populations were divided into the indicated memory subpopulations and the expression of α4β7 and α4β1 integrins determined.

The α4 integrins (α4β1, α4β7) are expressed by T and B cells^3,9^ and other leukocyte populations but whether certain cells may preferentially express either integrin heterodimer has not been demonstrated. Given potential redundancies of cell trafficking pathways during chronic inflammation^17^, we analyzed the surface expression of integrins α4β1 and α4β7 in the spleen, colonic LP, MLN, and peripheral blood of IL-10^−/−^ mice via mass cytometry. Our analysis demonstrated that T cells in spleen, LP, and blood predominantly expressed α4β1, while the B-cell lineage predominantly expressed α4β7^+^ (Fig. 1a, b, Supplementary Fig. 1a, b). The MLN was the exception, where CD8^+^ T cells were mostly α4β7^+^ and B cells expressing α4β1^+^ were most abundant (Supplementary Fig. 1a, b). We additionally examined the expression of α4 integrins on T-cell subsets (CD4^+^/CD25^+^ which include most Tregs, CD4^+^CD44^+^ effectors, CD4^+^/CD62L^+^ naive) as well as monocyte lineage cells (CD11c^+^/MHCII^+^ and CD14^+^/CD11b^+^). The three CD4 subsets and two myeloid lineage subsets examined predominantly express α4β1 (Supplementary Fig. 2). The derivation strategy for these analyses is shown in Supplementary Fig. 3a. To a lesser extent, other cell lineages also expressed α4β7^+^, including TCRγδ^+^, natural killer (NK), and myeloid cells (Supplementary Fig. 3b). Surface integrin expression does not appear to change during inflammation, as it was not different in T or B cells isolated from inflamed and uninflamed IL-10^−/−^ mouse colon (Supplementary Fig. 3c). In all, our findings support that most B cells in three of four immune sites examined predominantly express α4β7, whereas CD4^+^ and some monocyte lineage cells express predominantly α4β1, both in intestine and in the periphery.

### Integrin β7-deficient IL-10^−/−^ mice develop severe lethal colitis, which is associated with deficits in colonic B cell/ASC numbers and fecal IgA/M content

**Fig. 2:**
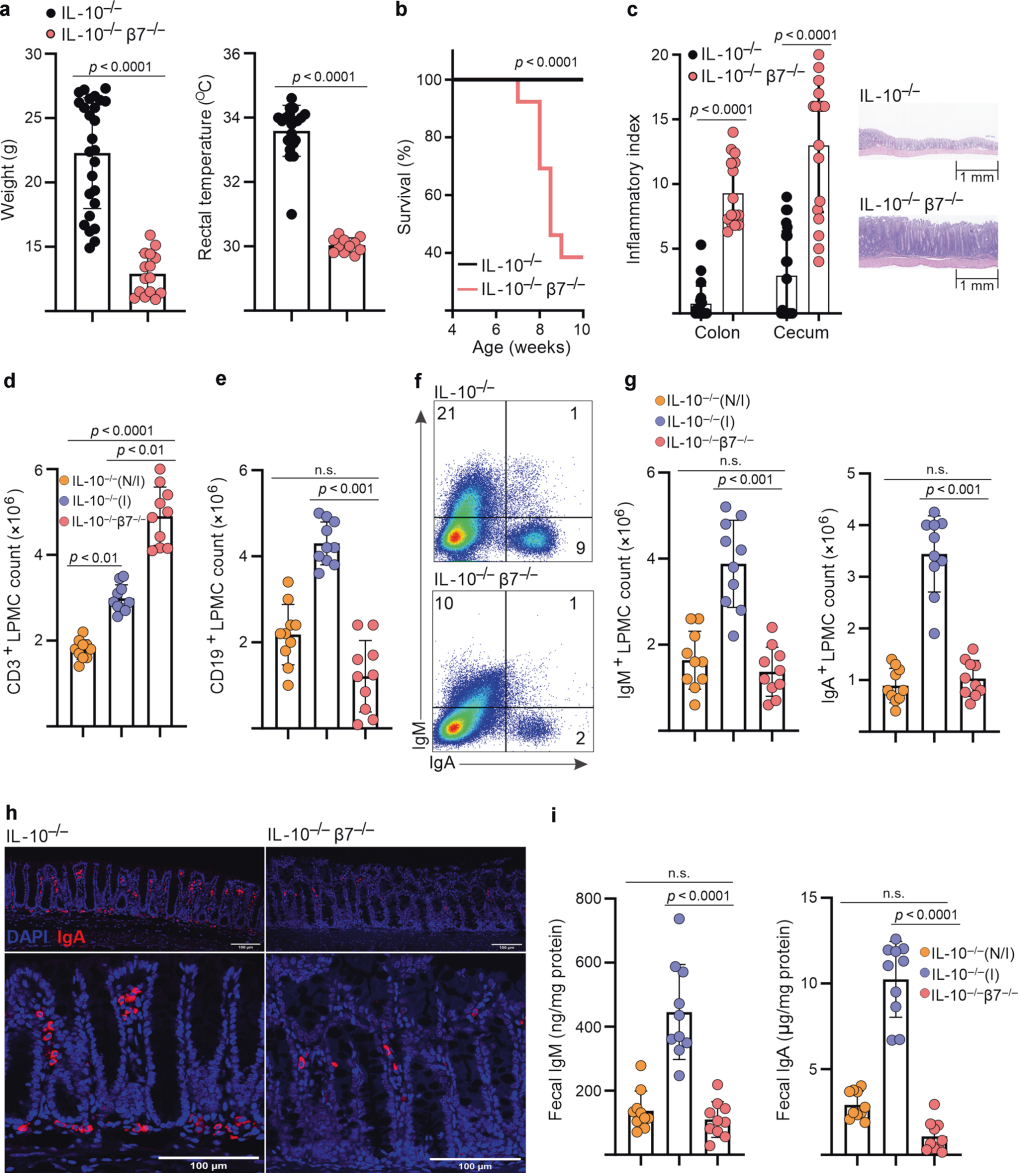
Integrin β7-deficient IL-10^−/−^ mice display deficits in colonic B cells populations, fecal immunoglobulin levels, and develop more severe colitis. **a** Weights, rectal temperatures and **b** survival curves of indicated strains. **c** Colonic and cecal histological indices and representative colonic pathology. **d** Colonic LP CD3^+^ T-cell and **e** CD19^+^ B-cell counts of indicated strains at 8–12 weeks of age, determined by flow cytometry (noninflamed (N/I, histological index ≤ 1), inflamed (I histological index > 3), freshly isolated LP cells were stained and events gated on live single cells. **f** Representative flow cytometry plots of colonic LP IgM^+^ and IgA^+^ cells. Events were gated on live, single, CD3^−^, CD19^−^ cells. **g** Colonic LP IgM^+^ and IgA^+^ cell counts of indicated strains. **h** Representative confocal immunofluorescence images demonstrating immunolocalization of colonic IgA^+^ ASC in the indicated mouse strains. **i** Fecal IgM and IgA of indicated mouse strains. Expression was normalized to total fecal protein. All data are presented as mean ± S.D, from *n* > 9 mice in each dataset. Each data point represents an individual mouse. Statistical significance was determined by ANOVA, followed by Tukey’s multiple comparison test (**a**, **b**, **d**, **e**) or Student’s *t* test (**g**–**i**).

Since our initial findings pointed to a predominant expression of α4β7 by B cells, we next examined its potential functional implications by assessing the effects of β7 deficiency on the colitic phenotype of IL-10^−/−^ mice. We compared the clinical phenotypes, immune cell distribution, and fecal Ig levels of β7-sufficient (IL-10^−/−^), with that of β7-deficient mice (IL-10^−/−^/β7^−/−^). IL-10^−/−^/β7^−/−^ mice developed worse colitis than their β7-sufficient counterparts, as indicated clinically by lower body weights, hypothermia, and decreased survival (Fig. 2a, b), as well as the nearly uniform development of rectal prolapse (Supplementary Fig. 4a). In addition, IL-10^−/−^/β7^−/−^ mice had significantly higher histological indices of colitis (Fig. 2c, Supplementary Fig. 4b), while the ileum was mostly uninvolved (Supplementary Fig. 4c). In our colony, β7-sufficient IL-10^−/−^ mice do not exhibit spontaneous colitis until after 16 weeks of age, which is often manifested clinically by rectal prolapse. Tissues were harvested once β7-deficient animals had lost 20% body weight (as per IRB regulations), which ranged from 7 to 12 weeks of age.

We next explored unexamined mechanisms that may underlie the increased severity of colitis in β7-deficient mice, by comparing the cellular composition of the colonic LP between the two strains (β7^−/−^ vs. β7^+/+^/IL-10^−/−^ mice). Given the variable penetrance of colitis in the strain, we separately analyzed noninflamed (N/I) and inflamed (I) mice, based on their histological colitis severity from 8 to 12 weeks of age, as determined by a pathologist in a blinded fashion, using a semiquantitative scoring system^18^. Our analysis showed that, upon development of intestinal inflammation, absolute numbers of both CD3^+^ and CD19^+^ cells increased in colitic (I) as compared with noncolitic (N/I) IL-10^−/−^ mice (Fig. 2d, e). Further increase in CD3^+^ T-cell counts was seen in IL-10^−/−^/β7^−/−^ mice corresponding to their more severe colitis (Fig. 2d). However, CD19^+^ cell counts remained at baseline in IL-10^−/−^/β7^−/−^ mice, despite their worse colitis (Fig. 2e). Further analysis revealed that the LP cell deficit was not limited to CD19^+^ B cells, but included IgM^+^ and IgA^+^ ASC (Fig. 2f–h). The LP ASC deficit was accompanied by lower fecal concentrations of immunoglobulins IgM, IgA (Fig. 2i), and IgG1-3 (Supplementary Fig. 4d), indicating a luminal pan-immunoglobulin deficit. T- and B-cell counts were mostly unchanged in spleen and MLN, in support of an intestinal-specific recruitment defect (Supplementary Fig. 4e). Taken together, these results suggest that influx of B-cell lineage cells into the inflamed intestinal mucosa is impaired in β7-deficient IL-10^−/−^ mice, whereas CD4^+^ T-cell entry remained unaffected. Therefore, β7 integrin deficiency appeared to lead to selective inhibition of B-cell intestinal trafficking, which results in low numbers of ASC at the colonic LP and a corresponding deficit of intraluminal immunoglobulins. These immunological effects are associated with significant worsening of colitis in β7-deficient mice, raising the possibility for a proinflammatory effect of the integrin deficit during the early stages of colitis induction, mediated by a fecal Ig deficit.

### Accelerated colitis in β7-deficient mice is not due to failure of mucosal regulatory mechanisms

**Fig. 3:**
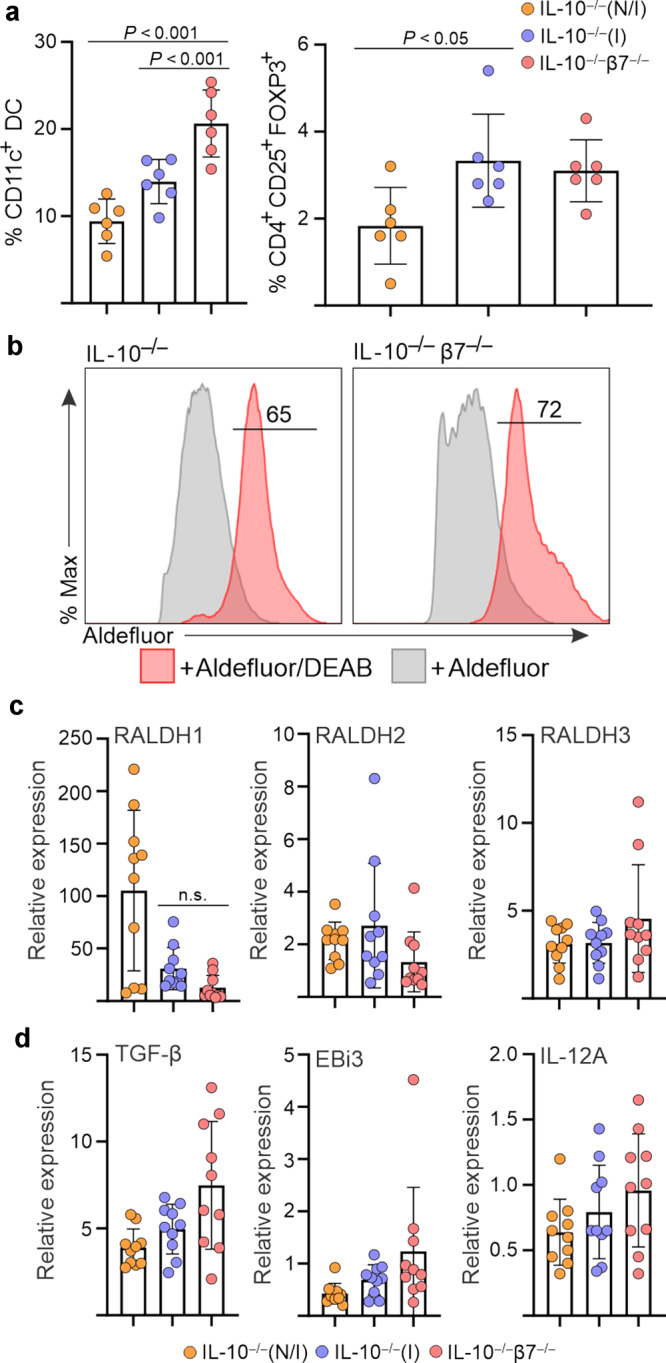
Dendritic cell and regulatory T-cell numbers, retinaldehyde dehydrogenase activity/mRNA expression, and regulatory B-cell-derived cytokines are preserved in IL-10^−/−^/β7^−/−^ mice. **a** Percentage of CD11c^+^ DC and CD4^+^CD25^+^FOXP3^+^ regulatory T cells within the colonic LP of the indicated mouse strains, as determined by flow cytometry (noninflamed (N/I), inflamed (I), as determined by histology). Events gated on live, single cells. **b** Aldefluor conversion within the colonic LP DC fraction isolated from the indicated strains. **c** Relative mRNA expression of RALDH1, RALDH2, and RALDH3 and **d** of regulatory cytokines and subunits within colonic tissues of indicated mouse strains. Expression of the respective genes was normalized to GAPDH expression. Each data point represents an individual mouse. Data are presented as mean ± S.D from *n* > 6 mice in each dataset. Statistical significance determined using ANOVA.

Next, in order to understand the mechanisms behind the acceleration of colitis in IL-10^−/−^/β7^−/−^ mice, we first tested the hypothesis that it was due to inadequate regulatory control at the intestinal mucosa. In fact, this was a plausible explanation, as β7^−/−^ mice, in addition to α4β7, lack αE(CD103)/β7 integrin also, which is involved in regulatory mechanisms. In particular, CD103 is expressed by a subset of intestinal DC^19^ that produce RA, and which are involved in the imprinting of a gut-homing phenotype on lymphocytes, induction of peripheral Tregs, and IgA class switching in ASC^2^. Accordingly, to test whether such regulatory mechanisms were defective in IL-10^−/−^/β7^−/−^ mice and responsible for the severe colitis phenotype, we measured the number of intestinal DC and Tregs, as well as the local production of Treg- or regulatory B-cell (Breg)-derived cytokines^20^ and the integrity of RA metabolism^2^. Nevertheless, we did not observe a shortfall of either colonic CD11c^+^ DC or Tregs (CD4/CD25/FoxP3^+^) (Fig. 3a). Similarly, aldefluor conversion, a surrogate indicator of retinaldehyde dehydrogenase (RALDH) activity (Fig. 3b), and RALDH1,2,3 mRNA expression (Fig. 3c) were normal in the LP of IL-10^−/−^/β7^−/−^ mice. Finally, the levels of T/B-cell regulatory cytokines (TGF-β, and IL-35 subunits EBI3 and IL-12A) (Fig. 3d) were equally expressed in β7-deficient and sufficient IL-10^−/−^ mice. Taken together, these findings indicate that impaired regulatory mechanisms mediated by CD103^+^ DC, Treg, or Bregs are preserved in IL-10^−/−^/β7^−/−^ mice and do not appear to play a major role on the aggravation of colitis, under the specific conditions of our IL-10^−/−^ mouse colony.

### MAdCAM-1 blockade worsened colitis in IL-10^−/−^ mice in association with depletion of colonic B cells and ASC, deficit in fecal IgA content, and generation of a dysbiotic microbiota

**Fig. 4:**
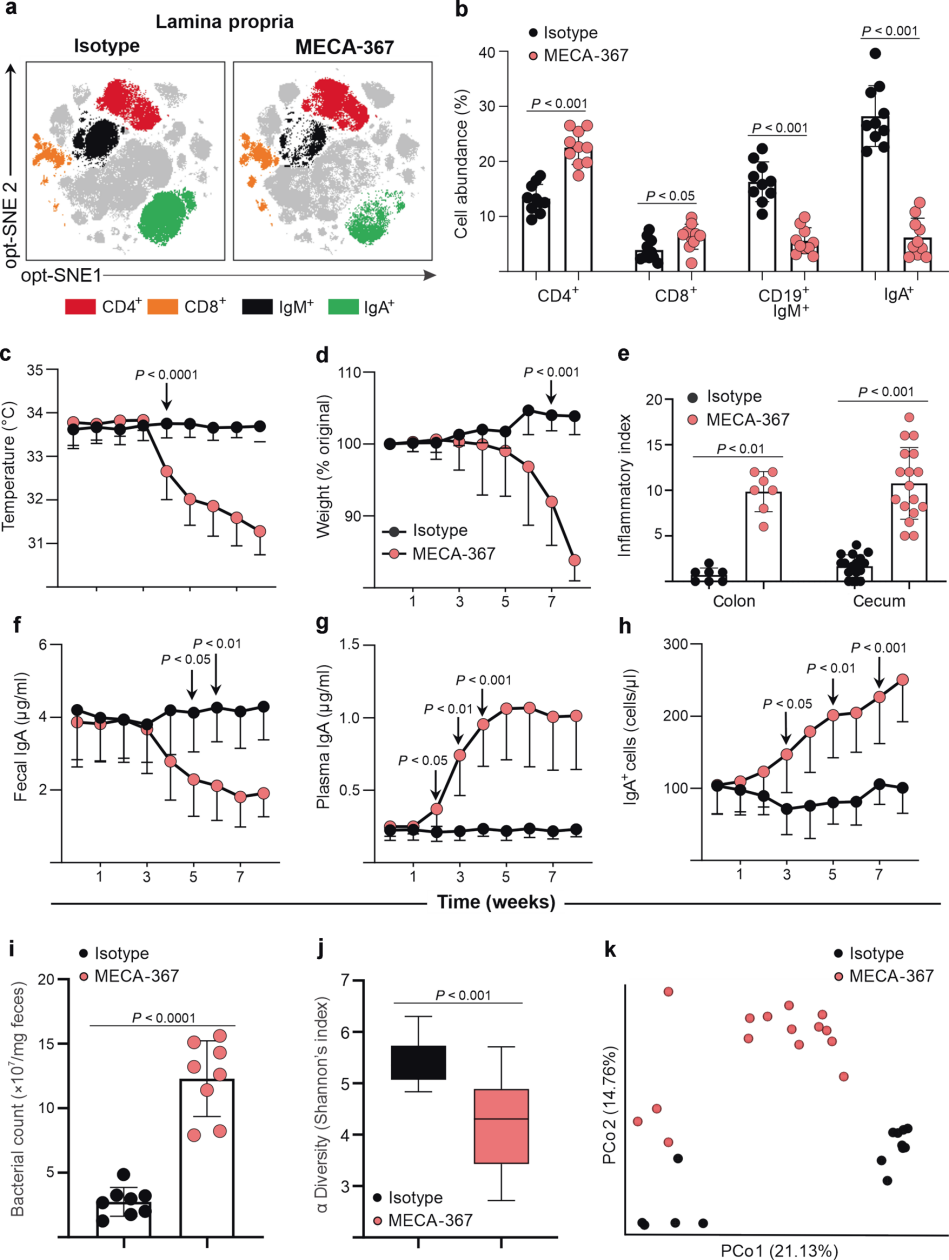
MAdCAM-1 blockade altered the percentage of B cell/ASC in colon and blood, reversed fecal, and plasma IgA levels, hastened colitis, and induced bacterial overgrowth and changes in microbiota composition. In IL-10^−/−^ mice, **a** mass cytometry opt-SNE analysis of concatenated colonic LPMC isolated from mice undergoing the indicated treatments, pregated on nucleated, live, CD45^+^ events. **b** Percentages of the indicated cell lineages within LPMC fractions of mice treated with MECA-367 or isotype control. **c** Serial rectal temperatures and **d** serial weights. **e** Colonic and cecal histological indices of treated mice at week 8. **f** Serial fecal IgA and **g** plasma IgA levels of treated IL-10^−/−^ mice. **h** Serial peripheral blood cell counts of IgA^+^ cells. **i** Fecal bacterial counts from IL-10^−/−^ mice treated with isotype or MECA-367, determined via RT-qPCR for 16S rRNA expression. **j** Alpha diversity (Shannon’s index) and **k** beta diversity principal coordinate analysis and of IL-10^−/−^ mice receiving indicated antibodies. All data are presented as mean ± S.D, from *n* > 10 mice in each dataset. Statistical significance was determined by Student’s *t* test (**b**) or two-way ANOVA, followed by Sidak’s multiple comparison test.

Mice that are deficient for β7 have a functional impairment that involves both αEβ7 and α4β7 integrins, either of which may be responsible for the worsening of colitis observed in IL-10^−/−^/β7^−/−^ mice. Thus, in order to dissect the roles of αEβ7 and α4β7 during chronic colitis, we treated IL-10^−/−^ mice with an antibody against the α4β7-specific ligand, MAdCAM-1 (MECA-367). In this way we specifically blocked α4β7-dependent traffic, leaving αEβ7-mediated pathways unaffected. Serial clinical and laboratory parameters were assessed prospectively and tissues were harvested after 8 weeks, when weights reached 80% of original at day 0. Mass cytometry analysis of the colonic LP showed that CD4^+^ and CD8^+^ T-cell populations increased, whereas CD19^+^IgM^+^ and IgA^+^ ASC were lower, compared with isotype-treated IL-10^−/−^ mice (Fig. 4a, b). Conversely, the percentage of CD19^+^IgM^+^, IgA^+^, and IgG^+^ ASC increased in the spleen, whereas CD4^+^ and CD8^+^ did not change (Supplementary Fig. 5a, b, e). Absolute numbers calculated with counting beads on a separate cohort using spectral cytometry confirmed these findings (Supplementary Fig. 5b). Clinically, mice developed progressive hypothermia starting at week 4 (Fig. 4c), followed by weight loss by week 6 (Fig. 4d). Inflammatory indices were higher in the colon and cecum of MECA-367-treated mice (Fig. 4e). Conversely, fecal IgA, IgM, and IgG levels decreased significantly by week 5 (Fig. 4f, Supplementary Fig. 5f) with a reverse increase in plasma IgA, IgM, and IgG beginning after 2 weeks, which preceded all other clinical and laboratory changes (Fig. 4g, Supplementary Fig. 5g). In agreement, IgA/M^+^ ASCs and CD19^+^ B cells increased in peripheral blood after 3 weeks (Fig. 4h, Supplementary Fig. 5c, d), whereas CD3^+^ T-cell counts were unaffected (Supplementary Fig. 5e). In our hands, MECA-367 neither affected the percentages of Tregs nor regulatory T/B-cell-derived cytokines (TGF-β, EBI3, IL12-A) (Supplementary Fig. 5h, i). In a separate experiment antibody blockade of the α4 integrin, which blocks both integrins α4β7 and α4β1 also induced weight loss, hypothermia and colitis in IL-10^−/−^ mice (Supplementary Fig. 6). Ongoing studies will attempt to define the relative functional dependence on these integrins for induction and maintenance of chronic colitis.

A relatively stable phenotype of unperturbed IL-10^−/−^ mice, rendered defective in α4β7:MAdCAM-1 interactions either via β7 deletion and anti-MAdCAM-1 antibody blockade led to a deficit of intraluminal IgA. Given the fact that IgA plays a crucial role for the maintenance of a homeostatic microbiota^21^, we hypothesized that inadequate control of the commensal microbiota leading to bacterial overgrowth/dysbiosis may contribute to the worsening of colitis. We, first, tested our hypothesis by examining fecal microbial counts and composition in cohoused IL-10^−/−^ mice treated with MECA-367 or isotype antibody via 16S qPCR and rRNA sequencing. Fecal bacterial counts were higher in MECA-367-treated mice, with lower α diversity (Fig. 4i) and altered β diversity by principal component analysis (Fig. 4j, k) compared with isotype-treated controls. Taken together, we show that MAdCAM-1 and α4 blockade recapitulate changes observed in β7-deficient mice thus, supporting a critical dependence of B cell/ASC on α4β7:MAdCAM-1 interactions for traffic to intestine and maintenance of luminal IgA.

### IgA deficiency recapitulates the accelerated phenotype observed in β7-deficient IL-10^−/−^ mice by inducing bacterial overgrowth and changes in microbiota composition

**Fig. 5:**
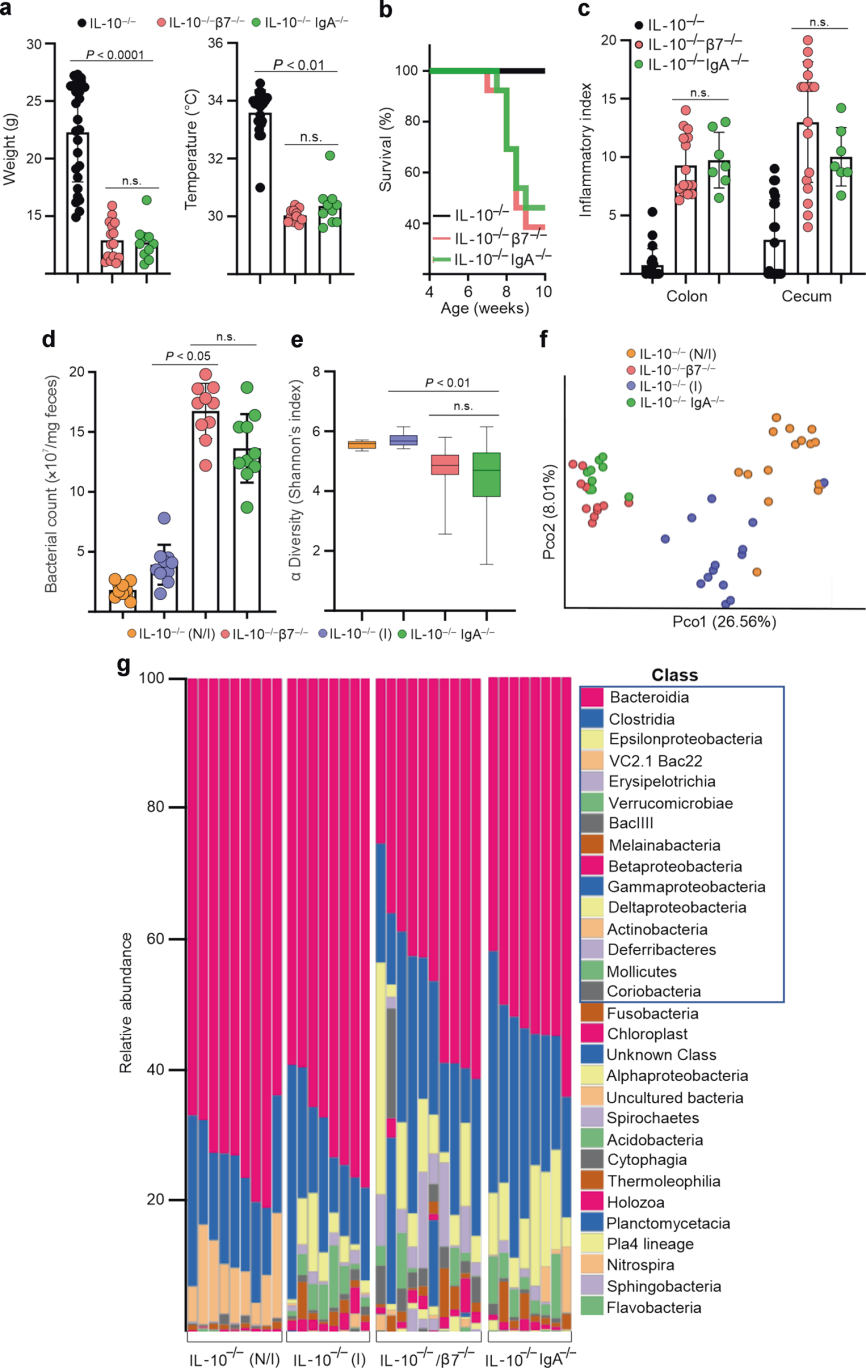
Disease phenotype and changes in microbiota observed in β7-deficient IL-10^−/−^ mice are recapitulated by IgA deficiency. **a** Weights, rectal temperatures, and **b** percent survival of indicated strains by 10 weeks of age. **c** Colon and cecum histological indices of disease severity of indicated genotypes at 8–12 weeks of age. **d** Fecal bacterial counts from indicated strains determined via RT-qPCR for 16S rRNA expression. **e** Fecal bacterial alpha diversity (Shannon’s index) and **f** beta diversity principal coordinate analysis of the indicated mouse groups. **g** Relative abundance of bacterial taxa at the class level in feces of the indicated mouse strains. All data are presented as mean ± S.D, from *n* > 10 mice in each dataset. Each data point represents an individual mouse. Statistical significance determined using one-way ANOVA, followed by Sidak’s multiple comparison test.

We then examined the potential association between the IgA deficit and aggravated colitis stemming from an α4β7-dependent B cell/ASC recruitment defect and by a congenital IgA deficit. We compared the clinical phenotypes and microbiota composition of IL-10^−/−^, IL-10^−/−^/β7^−/−^ mice, and IgA-deficient (IL-10^−/−^/IgA^−/−^) mice. Interestingly, IgA-deficient IL-10^−/−^ mice closely paralleled the phenotype of IL-10^−/−^/β7^−/−^ mice, inasmuch they developed more severe colitis than IL-10^−/−^ mice. This was reflected by lower weights, rectal temperatures, decreased survival (Fig. 5a, b), as well as worse inflammatory indices in both IL-10^−/−^/β7^−/−^ and IL-10^−/−^/IgA^−/−^ mice (Fig. 5c). Subsequently, we examined the effects on the microbiota of both the β7 and IgA deficits, both resulting in microbial overgrowth and changes in α and β diversity (Fig. 5d–f). Furthermore, although the development of colitis in IL-10^−/−^ mice led to baseline taxa redistribution, as compared with noninflamed littermates, both β7 and IgA deficiency resulted in additional changes in taxa, which were clearly distinct from inflamed and noninflamed mice (Fig. 5f). Interestingly, observed changes highly overlapped between IL-10^−/−^/β7^−/−^ and IL-10^−/−^/IgA^−/−^ mice (Fig. 5f, g) primarily represented by decreased *Bacteroides* and increased *Clostridiales*. Taken together, these data support a critical ASC dependency on α4β7/MAdCAM-1 interactions to migrate to the intestine and maintain luminal SIgA levels required for the maintenance of a homeostatic microbiota.

## Discussion

Understanding the mechanism of action of integrin-based therapeutics by defining their cellular targets may aid in the personalization of treatment options for IBD. We report, herein, that B cells at least in mice are uniquely dependent on α4β7 to access the colonic LP during chronic inflammation. We demonstrate that β7 deficiency and MAdCAM-1 blockade lead to colonic B cell/ASC deficits, fecal Ig deficit, and hastening of colitis, associated with major changes in microbiota composition, distinct from those induced by colitis alone. We also show that both the clinical phenotype and microbiota alterations resulting from defective or blocked α4β7/MAdCAM-1 interactions are recapitulated by IgA deficiency.

Here, we identify the B-cell lineage as the predominant cell population that expresses α4β7 integrin, both at the periphery and colonic LP of mice. In contrast, other lineages preferentially express α4β1. It has been proposed that expression of α4β7 and α4β1 dictates whether immune cells are destined for intestinal or peripheral sites^22^. Although subsets of T, B, NK, and myeloid cells and granulocytes were shown to express both integrins^9,10^, to our knowledge, our study is the first to examine the relative expression of each integrin across cell lineages in parallel, using unbiased high-dimensional techniques. Under homeostatic cell trafficking, α4β7 utilization by B cells represents the main pathway for B-cell extravasation into both GALT and LP^9,10,22–24^. In fact, the consequences of integrin β7 and MAdCAM-1 deficiencies are largely restricted to the B-cell lineage, including reduced Peyer’s patch size and deficits in B cell and IgA^+^ ASC populations within the LP^4,5^. On the other hand, B-cell recruitment during chronic intestinal inflammation has been less explored. This is of particular importance because chronic inflammation may modulate integrin/ligand expression patterns^25–29^, with both α4β7 and α4β1 integrins potentially mediating intestinal entry^26^. Such redundancy of inflammatory trafficking pathways may have important implications for the successful targeting of these molecules during IBD. Although our preliminary findings suggest differential expression of the α4 integrins by B cells and other leukocytes, we cannot reach any conclusions on the functional implications of this apparent dichotomy at this time.

To investigate the role of β7 integrins for cell entry into the colonic LP during colitis, we confirmed prior results that showed that β7 deficiency leads to significant worsening of colitis in this strain^30^.To understand whether B cells may participate on the immunological basis for such proinflammatory effect of β7 deficiency, we examined several different ideas. Firstly, β7^−/−^ mice lack not only α4β7 but also αEβ7 integrin. The latter is expressed by intraepithelial lymphocytes and by a subset of intestinal DC^19^ that produce RA, which imprints gut homing and regulatory phenotypes on T lymphocytes and induces IgA class switching in ASC^2^. Therefore, aggravated colitis in β7^−/−^ mice could be attributed to the absence of CD103^+^ DC and/or defective RA synthesis; nevertheless, we cannot confirm this to be the case as also supported by prior findings that showed that CD103-deficient DC are able to induce a gut-homing phenotype^31^, supporting an intact RA enzymatic machinery. On the one hand, no reduction in RA-synthetic enzyme expression, their activity, or Treg progeny was observed in β7^−/−^ mice. Furthermore, an anti-MAdCAM-1 antibody, which does not bind CD103, also hastened colitis in adult IL-10^−/−^ mice. Secondly, a compromise in regulatory mechanisms may also explain the worsening of colitis. Indeed a role of α4β7 for intestinal recruitment of Tregs has been proposed, and Treg blockade leads to worse colitis in IL-10^−/−^ mice^30^. However, under our unique experimental conditions, we neither observed a Treg or Breg deficit nor deficit of their regulatory cytokines in either IL-10^−/−^/β7^−/−^ or MECA-367 treated mice^20^.

Our findings allow us to propose an alternate or concurrent explanation for the aggravation of colitis in β7-deficient IL-10^−/−^ mice via intestinal dysbiosis due to a deficient ASC recruitment from PP leading to an intraluminal deficit of IgA and other immunoglobulins. These effects were also observed after anti-MAdCAM-1 treatment, and in IgA-deficient IL-10^−/−^ mice, pointing to a common downstream pathway of luminal SIgA deficit leading to dysbiosis. Our findings are in line with a critical role attributed to SIgA for the maintenance of a homeostatic microbiota^21^. Indeed, Fagarasan et al. showed a dramatic expansion of *Clostridia* in mice that lack activation-induced cytidine deaminase, an essential enzyme for class switch recombination and somatic hypermutation^32^. A similar expansion of *Clostridia* species was also detected in our study and recapitulated in IgA-deficient IL-10^−/−^ mice. Recently, minimal distortion of the resident microbiota composition was found in β7^−/−^ mice under homeostatic conditions, despite significant perturbation of the intestinal B-cell compartment^33^. However, it is possible that a baseline dysregulation of the microflora that is known to occur in inflamed IL-10^−/−^ mice^34^ is required for the accentuated effect of β7/IgA deficiency to take place. Nevertheless, whether dysbiosis or inflammation is the primary event remains to be determined. The effect of MAdCAM-1 blockade on the microbiota and colitis severity in IL-10^−/−^ cohoused siblings supports a causal effect, induced by the resultant luminal SIgA deficit, expansion of a dysbiotic flora, barrier breach, and possible systemic sepsis as reflected by hypothermia. In contrast, we observed broad distortion of microbiota composition following onset of colitis in β7-deficient IL-10^−/−^ mice, which may be reflective of impaired B-cell recruitment and inability to meet the demand for increased luminal SIgA, brought on by inflammation.

The increased severity of colitis in β7-deficient IL-10^−/−^ mice shown herein is in contrast with the majority of published studies in mouse models of colitis, which report anti-inflammatory effects of β7 deficit or anti-MAdCAM-1 antibody blockade^35^. In fact, the only exceptions so far have been the aggravated/lethal colitides that take place in β-deficient IL-10^−/−^ mice, a B-cell deficit (µMT/IL-10^−/−^ cross)^36^, MAdCAM-1 blockade^30^, and in colitis-prone Gαi2 mice after treatment with anti-α4 integrin antibody^37^. Those models are methodologically distinct from all others because the treatment course extended beyond 6 weeks. The timeline might be critical, because long-lived plasma cells are able to maintain fecal IgA levels for weeks, before the compromised plasmablast recruitment finally tilts the balance^38^. Of note, in our MAdCAM-1 blockade studies increases in serum IgA and peripheral B cell/ASC counts predated fecal IgA deficit by 2 weeks, suggesting that these cells accumulate in peripheral blood when unable to reach the intestine. Clinically, hypothermia also predated weight loss by 2 weeks, suggesting that the inflammatory process and potential barrier breach begins much earlier that it is manifested clinically by the uniformly used weight loss parameter. The MAdCAM-1 blockade model shows a dynamic process whereby effects on plasmablast recruitment result in converse effects in peripheral blood and LP, eventually leading to hypothermia, weight loss, and aggravated colitis. The timeline of integrin blockade may have implications for the mechanism of slow acting^39^ integrin-based therapies, due to potential differential effects on short-lived and long-lived cell subsets, the latter being less dependent on integrin-based recruitment for replenishment^6,7^. Interestingly, plasma and fecal IgA levels plateaued only after going through several weeks of increases/decreases, respectively, indicating that stabilization of the number of LP IgA^+^ ASC may take time. This may be explained by the existence of a subpopulation of B cells, which utilize alternative integrins for intestinal entry (e.g., α4β1), or may be due to long-lived plasma cells^38^ which may survive past the 8-week treatment protocol. The phenotype, integrin expression profile, and lifespan of IgA^+^ ASC that persist after sustained integrin/ligand blockade remains to be investigated.

Our study has potential translational implications for the treatment of patients with IBD. Firstly, it emphasizes the pathogenetic association and therapeutic potential of B cells in IBD, which have been largely underappreciated. This could be in part due to the failure of rituximab (anti-CD20 antibody) in UC^40^. Although it is known that CD20 is only expressed by a subset of cells of the B lineage. Similarly, most animal studies that examined the effects of trafficking blockade have focused almost exclusively on the effects on T cells, whereas the impact of the B cell/ASC/SIgA deficits has not been considered, or even dismissed as irrelevant^36^. Secondly, our study clearly highlights B cells as the principal α4β7-expressing population, uniquely dependent on α4β7:MAdCAM-1 for intestinal trafficking under inflammatory conditions. Human B cells also bind VDZ^10^ and the drug decreases B cells and lymphoid aggregates (inducible lymphoid follicles) in the intestine of HIV-1 infected individuals^41^. It also modulates the B-cell compartment in IBD^12,42^ and inhibits antibody responses to oral vaccination, even after a single dose^43^. In contrast, T cells express α4β7 and α4β1 heterogeneously^44^, whereas NK cells bind MAdCAM-1 poorly^22^. Those differential expression patterns of α4β7 and α4β1 integrins may have important implications for the clinical efficacy of drugs that target trafficking pathways. Currently, such therapies target either both α4 integrins (i.e., natalizumab), or β7 integrins (i.e., VDZ, etrolizumab), or MAdCAM-1 (i.e., ontamalimab). Thirdly, we describe the accumulation of B-cell subsets and Ig in blood after MAdCAM-1 blockade, similarly to splenic accumulation of IgA^+^ cells in β7^−/−^ mice^45^. Peripheral accumulation of α4β7^+^CD4^+^ T cells after VDZ treatment^26,46^ and MAdCAM-1 blockade^47^ has been reported previously. Those changes in peripheral cell frequency and serum immunoglobulin concentrations hold promise for the development of clinically useful biomarkers of response to anti-integrin therapies.

It remains difficult to reconcile the therapeutic effect of α4β7-MAdCAM-1 blockade seen in patients treated with VDZ or ontamalimab (anti-MAdCAM-1) with the hastening effect of a parallel intervention on IL-10^−/−^ colitis. First, evidence continues to accumulate that our understanding of the mode of action of VDZ and other anti-integrin therapies is incomplete. Second, the discrepancy could be attributed to a limited translational value of this specific model to human UC. However, a similar result was also reported in Gαi colitic mice^37^. Discrepancy with other preclinical immunoblockade studies could be additionally explained by the duration of treatment (less than 4 weeks in most studies)^35^. Of note is that we do not see a reduction in fecal IgA until week 5 of MAdCAM-1 blockade, as luminal SIgA is likely maintained by long-lived plasma cells that are less dependent on replenishment by IgA plasmablasts from blood. Alternatively, our preclinical observations might be more reflective of human IBD at its earliest stages (disease onset), triggered by loss of tolerance to bacterial antigens. This early stage of IBD is subclinical in humans and likely distinct from established clinical IBD, once the dysregulated immune process has become self-sustaining and less dependent on continuous bacterial antigenic stimulation. It is during this maintenance stage when patients present for medical care and are treated with VDZ, likely years after the onset of the initial dysregulated immune process.

Finally, while our data identify B cells as uniquely dependent on α4β7 for colonic entry in mice, it is clear that α4β7 is expressed and may be important for intestinal homing of other immune cell lineages in mice and humans. Schleier and colleagues reported that α4β7 is expressed by nonclassical monocytes, as defined by CD14 and CD16 coexpression (CD14+ CD16++) and that these cells increase in peripheral blood of VDZ-treated patients^13^. Indeed, VDZ modulates innate cell populations, importantly macrophages (increased M1, decreased M2). In another study however, adhesion of CD4^+^ T cells to MAdCAM-1 predicted responsiveness to VDZ in UC^46^. Thus, much remains to be learned regarding the definite mode of action of these drugs.

In conclusion, our data reiterate the critical dependence of B cells/ASC on α4β7/MAdCAM-1 interactions for intestinal recruitment, IgA production, and maintenance of a homeostatic microbiota. We propose that during chronic colitis in mice, most B cells are uniquely reliant on α4β7 pathways for intestinal recruitment, being unable to circumvent α4β7 deficiency, perhaps via α4β1-VCAM-1 interactions or other integrin pathways. This may have important implications for personalization of anti-integrin therapies as antibodies or small molecules that target both α4 integrins may be more efficacious in certain patient subsets (e.g., Crohn’s vs. UC). The relative dependence of different cell lineages on distinct integrins may define efficacy of a particular anti-integrin drug on individual patients.

## Materials and methods

### Mice

IL-10^−/−^ and integrin β7^−/−^ mice of the C57BL/6 background were obtained from The Jackson Laboratory (Stock nos.: 002251 and 002965, respectively) and bred in-house. IgA^−/−^ mice of the same background were a kind gift from Lars Eckmann^48^. Mice were cross-bred to homozygous state of the relevant gene mutations and genotype was confirmed by Transnetyx Inc (TN, USA).

For MAdCAM-1 and α4 blockade experiments, cohorts of 8-week-old IL-10^−/−^ mice were treated with either 100 μg of MECA-367 (anti-MAdCAM-1), PS-2 (anti-integrin α4), or rat IgG2a isotype control (both BioxCell, NH, USA), twice weekly, for a period of 8 weeks. Weekly weights, rectal temperatures, plasma, and fecal samples were collected throughout the course of the experiment. Cohorts of mice were euthanized and tissues harvested once MECA-367-treated mice had lost 20% of their original weight.

For mouse survival curves, mice were euthanized once reached 80% original body weight and this timepoint was recorded as death. Mouse colonic and cecal tissues were prepared for histological analysis by fixation in 10% neutral buffered formalin (Thermo Fisher Scientific, MA, USA), followed by 70% ethanol (VWR, PA, USA), embedded in paraffin, and cut into vertical 4-μm-thick sections. Tissue sections were stained with hematoxylin and eosin and histological assessment of inflammation was performed using a standardized semiquantitative scoring system^18^, by a trained pathologist (P.J.) blinded to the experimental conditions. Murine blood was obtained via submandibular bleed during the course of experiments or via cardiac puncture bleed at the time of tissue harvest. Serum was separated and stored at −80 °C.

### Cell isolation

Splenocytes were isolated via mechanical disruption of whole spleen, followed by red blood cell (RBC) lysis (RBC lysis buffer, BioLegend). Leukocytes were obtained from whole blood via two rounds of RBC lysis. Colonic LP cells were obtained as previously described^18^. In brief, colonic tissues were washed for 15 min at room temperature in 15 mL HBSS (Fisher Scientific), followed by three washes in HBSS + 1 mM EDTA (Hoefer Inc, MA, USA), followed by one wash in HBSS. Tissues were then subject to mechanical disruption and digestion for 45 min, in a solution containing complete RPMI + 1.5 mg/mL collagenase VIII and 1 μM DNase (both Sigma Aldrich). Resulting cell suspensions were filtered and utilized for downstream analyses.

### Mass cytometry antibody staining and barcoding

Cell suspensions were prepared for mass cytometry acquisition as previously described^49^, but with additional barcoding steps. In brief, isolated cells were resuspended in Maxpar cell staining buffer [CSB; Fluidigm, San Francisco, CA] and subsequently stained as follows: (1) viability staining using Cisplatin-195Pt [Fluidigm] for 5 min; (2) initial fixation step using Maxpar Fix I buffer [Fluidigm] for 20 min; (3) permeabilization and barcode labeling of each sample using the Cell-ID 20-Plex Pd Barcoding Kit [Fluidigm]. Following barcoding, cells from up to 20 different samples were combined into a single tube for further antibody labeling; (4) Fc receptor blockade using Mouse TruStain FcX [BioLegend, San Diego, CA] for 10 min; (5) surface antibody staining [Fluidigm; BioLegend, San Diego, CA, full antibody panels in Supplementary Table 1] for 30 min; (6) second fixation step using 1.6% formaldehyde [Methanol-free, Thermo Scientific] for 10 min; and (7) DNA-intercalator labeling using Maxpar Fix & Perm Buffer [Fluidigm] and Cell-ID Intercalator-Ir [Fluidigm], incubated at 4 °C overnight. Following overnight intercalator staining, samples were washed twice with CSB and stored at −80°C, in 90% FBS, 10% DMSO^49^. Where indicated, purified antibodies were conjugated with metal isotopes in-house, using antibody labeling kits [Fluidigm]. For barcoding distribution of samples, equal numbers of samples from each sample group were distributed across multiple barcode sets, to ensure comparable data acquisition and to minimize batch effects in the downstream data analysis.

### Mass cytometry sample acquisition and data processing

Prior to acquisition, cells were washed twice with Milli-Q water and resuspended in a 1:10 dilution of EQ Four Element Calibration Beads [Fluidigm] to a concentration of 0.5 × 10^6^ cells/mL. Samples were acquired using a CyTOF Helios [Fluidigm] and data normalized to mass bead signal using the Nolan lab Matlab software [GitHub, https://github.com/nolanlab]. Each Barcode set was fully acquired within 1 day of mass cytometry run time, and all barcode sets were acquired on sequential days.

### Mass cytometry data analysis

Following normalization, barcoded samples were debarcoded using the Nolan lab single-cell debarcoder tool [GitHub, https://github.com/nolanlab]. Mass cytometry data were analyzed using OMIQ [OMIQ, Inc] for biaxial gating, opt-SNE, FlowSOM, and edgeR^50^ analytical algorithms. Prior to t-distributed stochastic neighbor embedding [t-SNE] analysis, data were gated on nucleated, live, CD45^+^ events, then gated on the indicated populations of interest. Unless otherwise stated, opt-SNE analyses were conducted using 100,000 total events proportionally drawn from samples, with 1000 iterations and a perplexity value of 30. Where indicated, opt-SNE plots displayed were produced by concatenating multiple files into a single image, post analysis. FlowSOM analyses were conducted following opt-SNE analysis, using the Euclidean distance metric and consensus metaclustering with a comma-separated *k* value of 25. EdgeR analysis was performed on all identified clusters for each lineage described, and *P* values below 0.01 were utilized to determine significant differences. Summary graphs of cell abundances were produced using GraphPad Prism version 8 software [GraphPad Software Inc, La Jolla, CA].

### Flow cytometry

Viability staining was conducted using fixable aqua dead cell stain [Thermo Fisher Scientific] according to the manufacturer’s instructions. Fc receptor blockade was performed using TruStain FcX anti-mouse CD16/32 [both BioLegend]. Extracellular antibody staining was performed using the following antibodies: CD3 BV785, CD4 FITC, CD8 PE/Cy5, CD19 BV711, CD25 PE, integrin β7 BB515, CD49d PerCP/Cy5.5, CD11b BV605, CD11c BV711, IgA PE, IgM APC eFluor 780, IgG BV421, and MHCII PerCP/Cy5.5. Following staining, cells were fixed using the BD stabilizing fixative solution [BD Biosciences]. Where required, intracellular staining was performed using the FOXP3 Fix/Perm Buffer Set (BioLegend), according to the manufacturer’s instructions, as well as anti-mouse FOXP3 BV421 (BioLegend). Cells were acquired on a 16-color BD LSRII [BD Biosciences]. Where indicated, cells were acquired alongside AccuCheck counting beads [Thermo Fisher Scientific] for absolute cell counts. Where applicable, cells were incubated with Aldefluor reagent (Stemcell Technologies, Vancouver, BC, Canada) to identify cells that express high levels of aldehyde dehydrogenase (ALDH). The activated reagent, BODIPY-aminoacetaldehyde, is a fluorescent substrate for ALDH that diffuses into viable cells and is converted to BODIPY-aminoacetate and retained intracellularly. The fluorescent reaction product is proportional to the ALDH activity. Diethylaminobenzaldehyde is a specific inhibitor of ALDH used to control for background fluorescence. Flow cytometry data were analyzed using FlowJo version 10 (Treestar, OR, USA).

### Soluble immunoglobulin measurements

Fecal and plasma murine immunoglobulin levels were determined using the Mouse Isotyping Panel 1 Kit (Meso Scale Discovery, MD, USA). Fecal pellets were vortexed in 1 mL of PBS for 10 min, centrifuged at 12000 × *g* for 10 min twice, and supernatant collected for Ig level determination. Plasma was diluted at 1:10,000 and fecal supernatant was diluted at 1:100 for analysis. Fecal Ig levels were normalized per mg of total fecal protein content, as determined by Bradford Protein Assay (Bio-Rad, CA, USA). Plates were read using a MESO QuickPlex SQ 120, and analyzed using the MSD workbench software.

### Immunofluorescence

Four-micrometer-thick paraffin sections were deparaffinized, rehydrated, and permeabilized with Triton X-100 0.01% in PBS for 1 h at room temperature, blocked with 1% BSA in PBS for 1 h, then stained with 2.5 μg/mL anti-mouse IgA primary antibody (556969; BD Pharmingen, NJ, USA) in blocking buffer (1% BSA in PBS) for 18 h at 4 °C. Sections were subsequently incubated with 2 μg/mL Alexa Fluor 594 goat anti-rat IgG (H + L) secondary antibody (A-11007, Thermo Fisher Scientific, MA, USA) for 2 h at 4 °C. After washing, specimens were mounted with ProLong™ Gold Antifade Mount with DAPI (P36935, Thermo Fisher Scientific, MA, USA) and stored at 4 °C. Image acquisition was performed with a Zeiss LSM780 confocal microscope (Zeiss, Jena, Germany) equipped with a 40×/1.3 NA EC Plan-Neofluar oil objective. High-resolution images of the whole sample were taken through a tile scan.

### Microbiota analysis

Fecal samples were stored at −80 °C until use. DNA was extracted using the QIAamp DNA Stool Mini Kit (Qiagen, Germany), according to the manufacturer’s guidelines. V4 fragments of the 16S rRNA gene were sequenced using an Illumina MiSeq platform. Sequencing datasets were analyzed using the QIITA online analysis platform. Sequencing files were demultiplexed, trimmed to 150 base pairs, deblurred, and operational taxonomic unit assigned using the Silva-119 database. Data were rarified, alpha diversity determined using Shannon’s index, and beta diversity determined using the Bray–Curtis dissimilarity, followed by principal coordinate analysis.

### Real-time qPCR

Colonic tissues were harvested and stored at −80 °C in RNAlater solution (Thermo Fisher Scientific). RNA was isolated from colonic tissues using the RNeasy Mini Kit (Qiagen, Germany). cDNA was generated using the High Capacity cDNA Reverse Transcription Kit (Applied Biosystems, CA, USA). Gene expression was examined using the following Taqman probes (all Thermo Fisher Scientific): TGFβ (Tgif1, Mm01227699_m1), EBI3 (Mm00469294_m1), IL-12A (Mm00434169_m1), RALDH1 (Aldh1a1, Mm00657317_m1), RALDH2 (Aldh1a2, Mm00501306_m1), and RALDH3 (Aldh1a3, Mm00474049_m1), and normalized to GAPDH (Mm99999915_g1).

### Statistics

Statistical analysis was performed using GraphPad Prism 8 software [GraphPad Software Inc]. For comparisons of two groups, the Student’s *t* test was utilized. For comparison of multiple groups, ANOVA was utilized, followed by Tukey’s multiple comparison test. Descriptive statistics are displayed as mean ± standard deviation in all figures. Significance is defined as *P* values of <0.05, resulting statistical significances of difference are indicated in figures as *P* < 0.05, *P* < 0.01, *P* < 0.001, and *P* < 0.0001, and n.s represents comparisons which are not statistically significant in difference.

## Supplementary Information

Supplemental material
