# Supplementary material for: Antibody secreting cells are critically dependent on integrin α4β7/MAdCAM-1 for intestinal recruitment and control of the microbiota during chronic colitis
Source: Mucosal Immunol. 2021 Aug 25;15(1):109–19. doi: 10.1038/s41385-021-00445-z (PMC8732264; doi:10.1038/s41385-021-00445-z)
Supplement: Supplementary file 1 — Supplemental material [file 41385_2021_445_MOESM1_ESM.pdf]

Supplemental Material

Antibody secreting cells are critically dependent on integrin α4β7/MAdCAM-1 for intestinal recruitment and control of the microbiota during chronic colitis

Christopher J. Tyler[1,2], Mauricio Guzman[1,2], Luke R. Lundborg[1,2], Shaila Yeasmin[1,2], Nadia Zgajnar[1,2], Paul Jedlicka[3], Giorgos Bamias[4] and Jesús Rivera-Nieves[1,2]

[1]San Diego VA Medical Center, San Diego, CA.

[2]Division of Gastroenterology, University of California San Diego, La Jolla, CA.

[3]Department of Pathology, University of Colorado Denver

[4]GI Unit, 3rd Academic Department of Internal Medicine, National and Kapodistrian University of Athens, Sotiria Hospital, Athens, Greece.

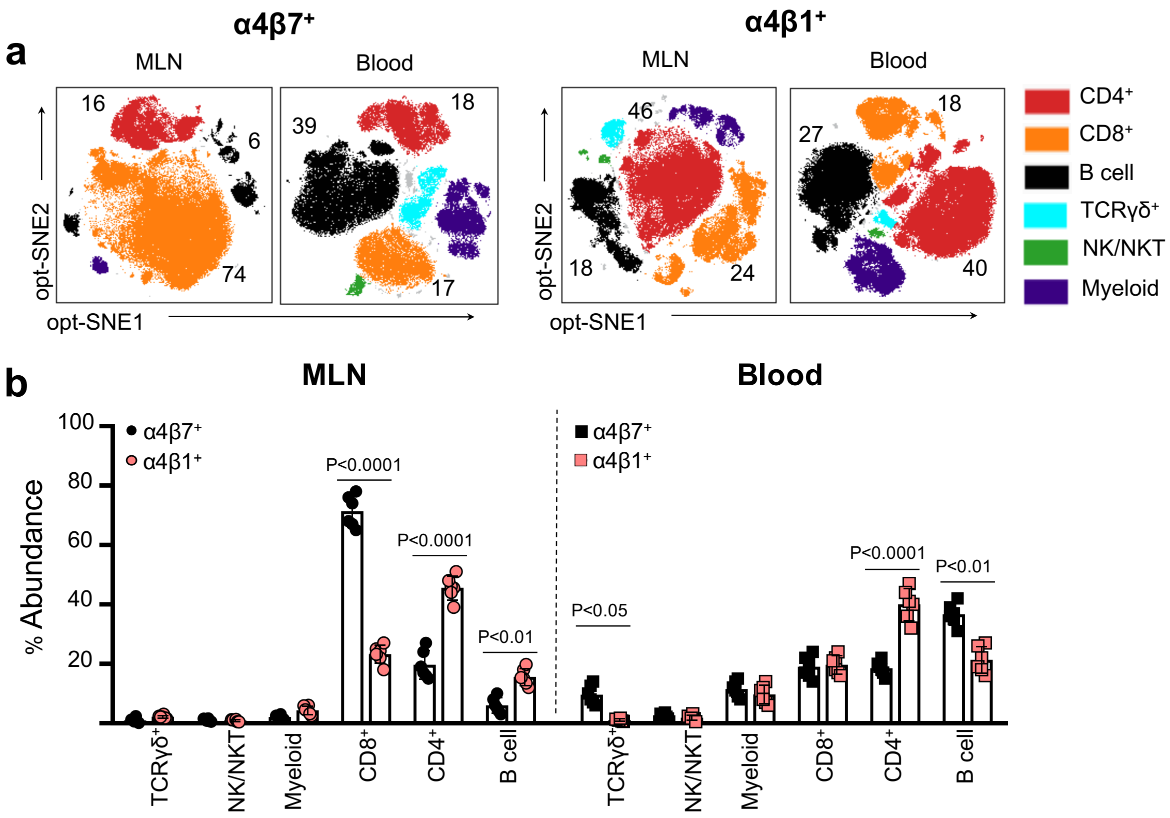

Supplemental Figure 1. B cells express predominantly α4β7 integrins except at MLN. (a) Cellular distribution of integrins α4β1 and α4β7 among the major leukocyte lineages within the MLN and peripheral blood. Cells were pre-gated on live, CD45$^+$ cells, followed by opt-SNE analysis. Cell populations including CD4$^+$ (CD4$^+$ T cells), CD8$^+$ (CD8$^+$ T cells), CD19$^+$/IgA$^+$/IgG$^+$ (B cells), TCRγδ$^+$ (γδ T cells), CD56$^+$ (NK/NKT), or MHCII$^+$/CD11c$^+$/CD11b$^+$ (myeloid) cells are highlighted. **(b)** Percentages of α4β1- and α4β7-expressing cells within the indicated cell lineages isolated from MLN and blood. Each data point represents a single mouse. All data are presented as mean ± S.D, from n>6 mice in each dataset. Statistical significance determined using ANOVA, followed by Tukey's multiple comparison test.

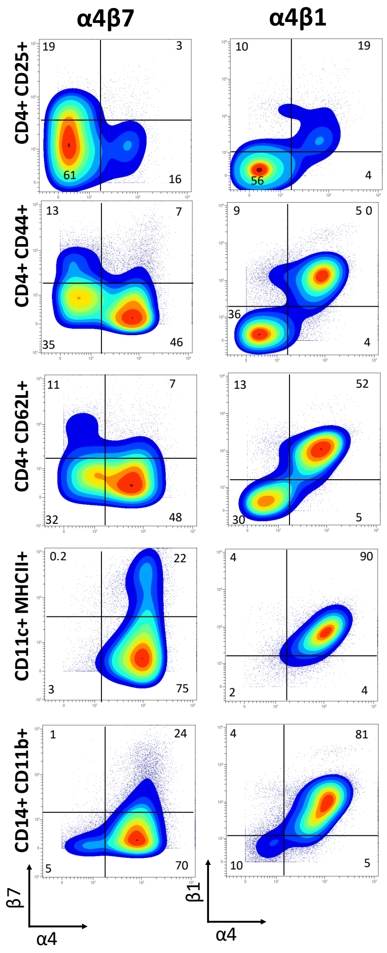

Supplemental Figure 2. CD4 subsets and myeloid lineage subsets predominantly express integrin α4β1 in the colonic LP. Representative pseudo-color plots of α4, β7 and β1 expression of the indicated cell subsets in the colonic LP of IL-10$^{-/-}$ mice. Cells were pre-gated on live, CD45^{+} cells, followed by two-dimensional analyses of mass cytometry data.

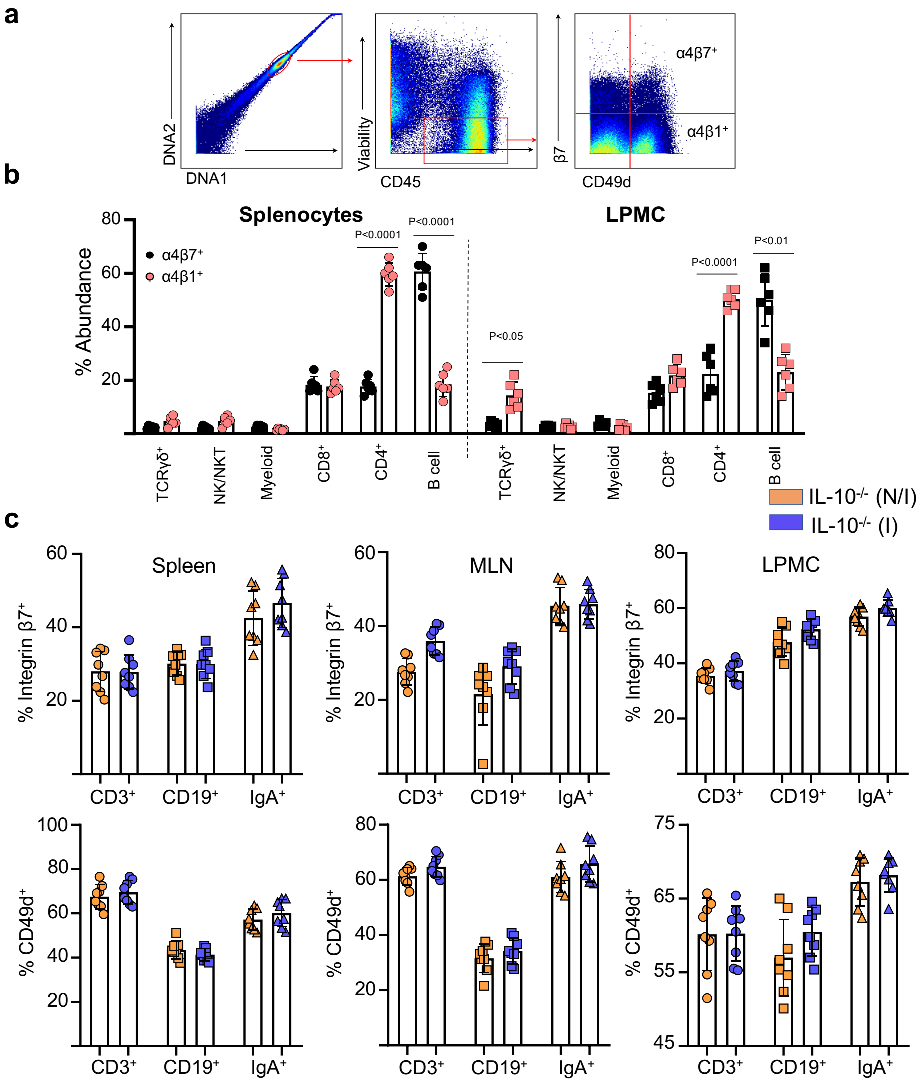

Supplementary Figure 3. Gating strategy and percentage of cell subsets expressing α4 integrins in IL10-/- mice and comparison of integrin expression in mice with and without colitis. (**a**) Gating strategy for the opt-SNE analysis displayed in Figure 1 and Supplemental Figures 1,2,3. (**b**) Summary data displaying lineages represented within murine α4β7 and α4β1 cellular events, derived from either splenocytes and colonic LPMC. (c) α4 and β7integrin expression in IL-10$^{-/-}$ mice with (inflamed (I) and without colitis (NI). Each data point represents a single mouse. Error bars display standard deviation of mean. Statistical significance determined using ANOVA, followed by Tukey's multiple comparison test.

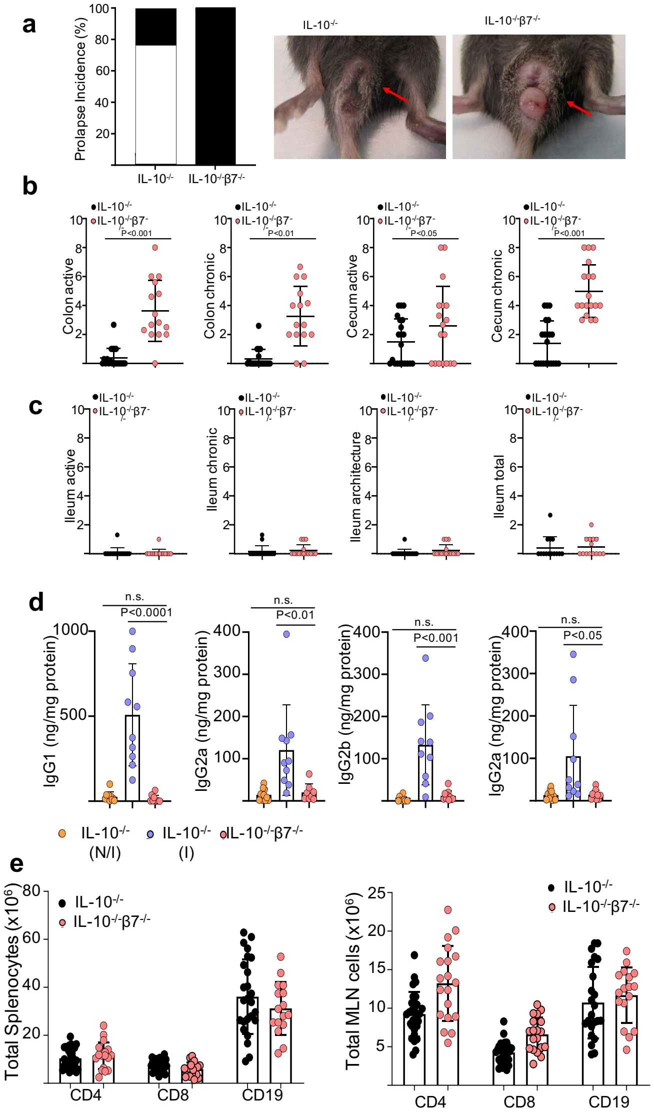

Supplementary Figure 4. Colonic and ileal inflammatory subscores, fecal IgG and cell counts of IL-10$^{-/-}$ and IL-10$^{-/-}$β7$^{-/-}$ mice. (**a**) Incidence of rectal prolapse among indicated mice at 12-weeks-of-age and representative images. (**b, c**) Active and chronic inflammatory indices of colon and cecum and ileum of indicated mice at 8- to 12-weeks-of-age. (**d**) Fecal IgG1, IgG2a, IgG2b, and IgG3 levels of the indicated mouse strains. Immunoglobulin levels were normalized to total fecal protein concentration. (**e**) Absolute cell counts of the indicated cell lineages within spleen and MLN of the indicated mouse strains. Each data point represents an individual mouse. Error bars display mean and standard deviation of datasets. Statistical significance determined using Student's t-test or ANOVA.

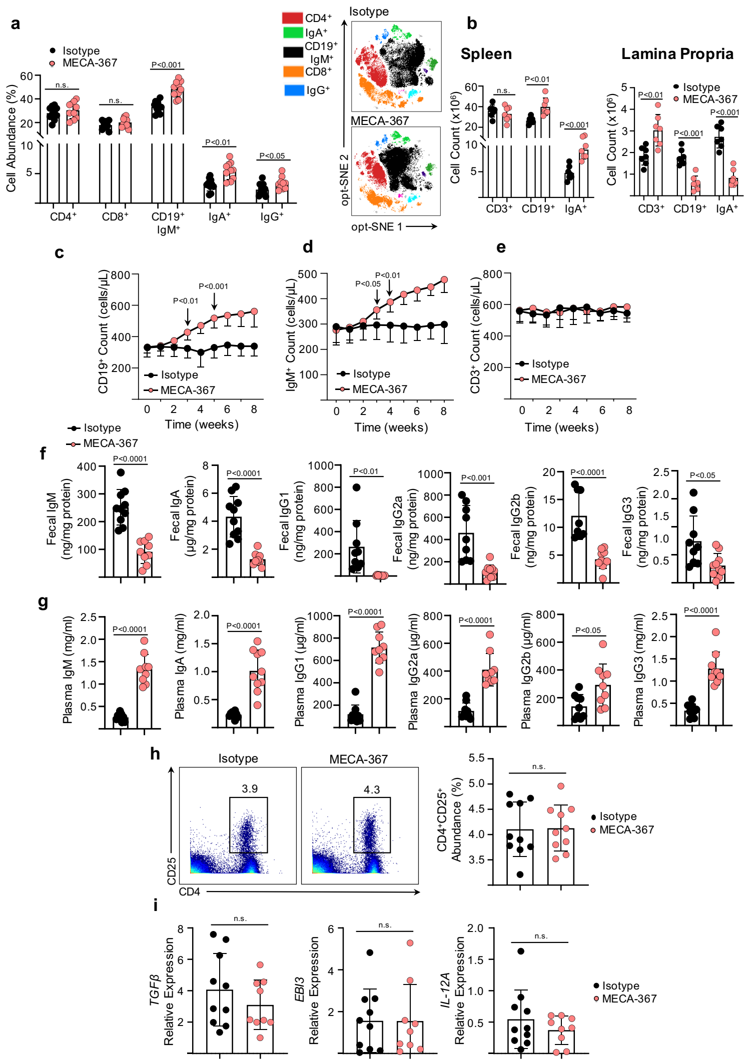

Supplementary Figure 5. Additional MAdCAM-1 blockade experimental data. (**a**) Percentage of indicated cell types from spleen of treated IL-10$^{-/-}$ mice, pre-gated on nucleated, live, CD45^{+} events. Samples (n=10) were concatenated to generate the opt-SNE plot. (**b**) Absolute cell counts of the indicated cell populations derived from spleen or colonic LP of mice after indicated treatments. (**c-e**) Peripheral blood cell counts of (**c**) CD19^{+}, (**d**) IgM^{+}, and (**e**) CD3^{+} cell populations of mice undergoing the indicated treatments. (**f**) Fecal and (**g**) plasma levels of IgM, IgA, IgG1, IgG2a, IgG2b, and IgG3, derived from IL-10$^{-/-}$ mice treated with either isotype or MECA-367 for 8 weeks. (**h**) Representative biaxial mass cytometry plots and percentages of CD4^{+}CD25^{+} T cells within the colonic lamina propria of IL-10$^{-/-}$ mice treated with either isotype or MECA-367 for 8 weeks. (**i**) mRNA expression of regulatory cytokines in colon of IL-10$^{-/-}$ mice receiving indicated treatments for 8 weeks, as determined by RT-qPCR. Error bars display mean and standard deviation of datasets. Each data point represents an individual mouse. Statistical significance determined using student's t-test.

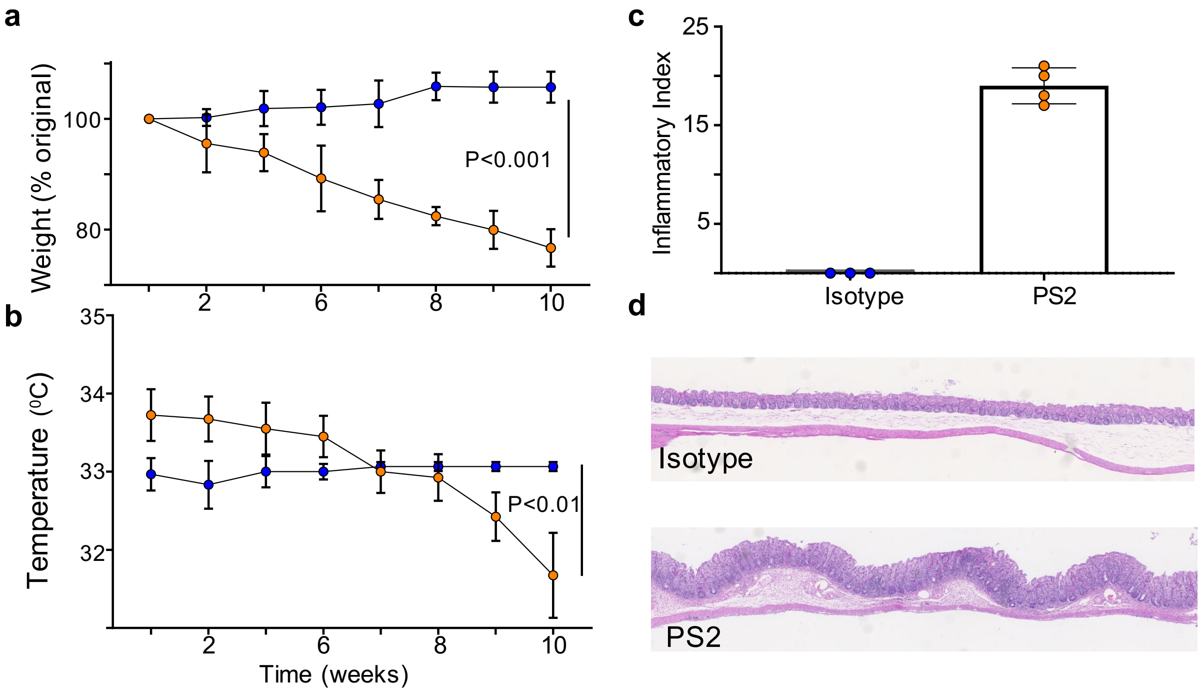

Supplemental Figure 6. Integrin α4 blockade hastened colitis in IL10-/- mice. (**a**) Serial weights (**b**) rectal temperatures, (**c**) colonic inflammatory indices of IL-10$^{-/-}$ mice treated for 10 weeks with isotype(●)or MAb PS-2 (anti-α4(●). (**d**) Representative images. All data are presented as mean ± S.D. from values obtained from 3-4 mice per cohort. Statistical significance was determined by student's t-test.

mAb	Conjugate	Company	Volume/Test	Clone	Product Number
CD45	89Y	Fluidigm	1ul	30-F11	3089005B
Barcode 1	102Pd	Fluidigm	-	-	-
Barcode 2	104Pd	Fluidigm	-	-	-
Barcode 3	105Pd	Fluidigm	-	-	-
Barcode 4	106Pd	Fluidigm	-	-	-
Barcode 5	108Pd	Fluidigm	-	-	-
Barcode 6	110Pd	Fluidigm	-	-	-
CD19	CD112 (Qdot 655)	Thermofisher	0.5ul	6D5	Q10379
CD20	113Cd	BioLegend	1ul	SA271G2	152102
CD14	114Cd	BioLegend	1ul	Sa14-2	123321
CD44	141Pr	BioLegend	0.5ul	IM7	103051
CXCR5	142Nd	Fluidigm	1ul	L138D7	3142015B
CD69	143Nd	Fluidigm	1ul	H1.2F3	3143004B
CD16/32	144Nd	Fluidigm	1ul	93	3144009B
CD45Rb	145Nd	Fluidigm	0.5ul	C363.16A	3145012B
CD5	146Nd	Fluidigm	0.5ul	537.3	3146012B
CD29	147Sm	BioLegend	1ul	HMB1-1	102202
CD11b	148Nd	Fluidigm	0.25ul	M1/70	3148003B
CD4	149Sm	BioLegend	2ul	RM4-5	100561
IgD	150Nd	Fluidigm	1ul	11-26c.2a	3150011B
CD49d	151Eu	N/A	1ul	PS/2	3151016B
CD24	152Sm	Fluidigm	1ul	145-2C11	3152004B
NKp46	153Eu	Fluidigm	0.5ul	29A1.4	3153006B
CXCR3	154Sm	BioLegend	2ul	CXCR3-173	126502
CD103	155Gd	BioLegend	2ul	2E7	121402
CCR6	156Gd	Fluidigm	1ul	292L17	3156016A
CCR9	158Gd	Origene	2ul	9B1	TA320235 Ccr9
CXCR4	159Tb	Fluidigm	1ul	L276F12	3159030B
IgA	160Gd	BioLegend	1ul	RMA-1	407002
IgM	161Dy	BioLegend	0.5ul	RMM-1	406527
Integrin B7	162Dy	Fluidigm	1ul	FIB504	3162026B
CCR10	163Dy	R&D	1ul	MAB2815	MAB2815
CD62L	164Dy	Fluidigm	1ul	MEL-14	3164003B
CD80	165Ho	BioLegend	1ul		
CD279 (PD-1)	166Er	BioLegend	2ul	RMP1-30	109113
TCRgd	167Er	BioLegend	0.5ul	GL3	118101
CD8	168Er	Fluidigm	1ul	536.7	3168003B
CD25	169Tm	BioLegend	2ul	3C7	101902
CD161	170Er	Fluidigm	1ul	PK136	3170002B
CD38	171Yb	Fluidigm	1ul	90	3171007B
IgG	172Yb	Fluidigm	1ul	Poly	3172001G
CD27	173Yb	BioLegend	2ul	LG.3A10	124202
I-A/I-E (MHCII)	174Yb	Fluidigm	0.25ul	M5/114.15.2	3174003B
CD127	175Lu	Fluidigm	1ul	A7R34	3175006B
CD11a	176Yb	BioLegend	1ul	I21/7	153102
CD11c	209Bi	Fluidigm	1ul	N418	3209005B
DNA1	191Ir	Fluidigm	-	-	-
DNA2	193Ir	Fluidigm	-	-	-
Cisplatin	195Pt	Fluidigm	-	-	-

Supplemental Table 1. Mass cytometry antibody panel
